# Clinical Relevance of Loss of 11p15 in Primary and Metastatic Breast Cancer: Association with Loss of PRKCDBP Expression in Brain Metastases

**DOI:** 10.1371/journal.pone.0047537

**Published:** 2012-10-31

**Authors:** Harriet Wikman, Bettina Sielaff-Frimpong, Jolanthe Kropidlowski, Isabell Witzel, Karin Milde-Langosch, Guido Sauter, Manfred Westphal, Katrin Lamszus, Klaus Pantel

**Affiliations:** 1 Institute of Tumor Biology, University Medical Center Hamburg-Eppendorf, Hamburg, Germany; 2 Department of Gynecology, University Medical Center Hamburg-Eppendorf, Hamburg, Germany; 3 Institute of Pathology, University Medical Center Hamburg-Eppendorf, Hamburg, Germany; 4 Department of Neurosurgery, University Medical Center Hamburg-Eppendorf, Hamburg, Germany; National University of Ireland Galway, Ireland

## Abstract

The occurrence of brain metastases among breast cancer patients is currently rising with approximately 20–25% incidence rates, underlining the importance of the identification of new therapeutic and prognostic markers. We have previously screened for new markers for brain metastasis by array CGH. We found that loss of 11p15 is common among these patients. In this study, we investigated the clinical significance of loss of 11p15 in primary breast cancer (BC) and breast cancer brain metastases (BCBM). 11p15 aberration patterns were assessed by allelic imbalance (AI) analysis in primary BC (n = 78), BCBM (n = 21) and metastases from other distant sites (n = 6) using six different markers. AI at 11p15 was significantly associated with BCBM (p = 0.002). Interestingly, a subgroup of primary BC with a later relapse to the brain had almost equally high AI rates as the BCBM cases. In primary BC, AI was statistically significantly associated with high grade, negative hormone receptor status, and triple-negative (TNBC) tumors. Gene expression profiling identified *PRKCDBP* in the 11p15 region to be significantly downregulated in both BCBM and primary BC with brain relapse compared to primary tumors without relapse or bone metastasis (fdr<0.05). qRT-PCR confirmed these results and methylation was shown to be a common way to silence this gene. In conclusion, we found loss at 11p15 to be a marker for TNBC primary tumors and BCBM and *PRKCDBP* to be a potential target gene in this locus.

## Introduction

Breast cancer (BC) is the most common malignancy in women and a major cause of morbidity and mortality. Metastatic breast cancer remains essentially incurable, with mortality and life quality impairments being especially high in patients who develop brain metastases. Survival for patients with brain metastases treated with whole-brain radiation therapy is typically only 4–6 months [1]. Approximately 15–20% of all breast tumors metastasize to the brain, with increasing incidence rates mainly due to more efficient treatment of primary tumors and increased use of sensitive detection methods [2].

In primary breast cancer, several chromosomal regions have been identified as being involved in tumor initiation and progression [3], [4], [5]. Many of these loci have further been linked with metastasis or aggressive behavior. However, only very few studies have actually investigated the chromosomal aberration patterns in distant metastases [6], [7], [8], [9]. Metastasis suppressors and oncogenes might not be identified when investigating primary tumors as they do not play an important role in primary tumor growth instead they are important for the dissemination or out growth of metastases.

Chromosomal deletion and allelic loss is a common feature of the malignant progression of human tumors, and a high rate of chromosomal loss at a certain region usually indicates the presence of a tumor suppressor gene. Fine mapping analysis by e.g. loss of heterozygosity (LOH) can be further used for the identification of putative tumor suppressor genes residing at that particular chromosomal region. In a previous study we compared the chromosomal aberration patterns between primary BC and breast cancer brain metastases (BCBM) by array CGH analysis. We identified 5 chromosomal regions that were significantly more often aberrated in the BCBM patients. Loss of 11p15 was found deleted in 71% of the brain metastases, whereas only 13% deletions were detected in early stage primary tumors [10].

Homozygous or hemizygous loss at the chromosomal region 11p15 has been observed in many cancer types including lung, pancreas, and bladder cancers [11], [12], [13]. Also in primary BC loss of 11p has been associated with invasiveness and worse prognosis [14]–[19], however until now no clear target gene within this rather small region has been identified.

In this study we investigated the role of 11p deletion in brain metastasis formation and identified *PRKCDBP* (also known as *hSRBC*) as a potential target gene for this region.

## Materials and Methods

### Sample Collection and Clinical Data

Samples were collected from patients who underwent surgical resection at the University Medical Center, Hamburg-Eppendorf, Germany. All primary tumor samples were collected from patients operated between 1997 and 2009 and the brain metastases samples were collected from patients operated between 2003 and 2009. Clinical data are summarized in Table S1. For microsatellite analysis 78 primary BC, 21 BCBM samples, and six other metastatic samples were analyzed. Ten of the primary tumor patients suffered a brain relapse during the follow up (FUP) period (relapse mean: 33.6 months, range: 4.9–73.1), and 20 patients had a relapse to other organs than brain (relapse mean: 30.1 months, range: 3.0–63.6). 44 of the primary tumor patients remained relapse free (FUP mean: 52.4 months, range: 7.7–97.9). Four matched pairs of BCBM and primary BC was available. In six patients no FUP information was available. For quantitative RT-PCR analyses RNA was available from 15 BCBM and 23 primary BC samples, whereas for MSP analyses 16 BCBM and 13 primary tumor samples were available. All sample donors gave written informed consent to biological research into their samples as approved by the ethics committee of the chamber of physicians, Hamburg, Germany. All clinical investigation have been conducted according to the principles expressed in the Declaration of Helsinki.

### Microsatellite analysis

An 11.5 Mbp region at 11pter-p11.3 (chr11:335,808–11,809,431) was analyzed by microsatellite analysis (allelic imbalance, AI) using six markers. If necessary manual microdissection was performed in order to obtain a tumor cell content of at least 70% [20]. Tumor DNA was isolated from either fresh frozen samples (n = 94) using the QIAmp DNA MicroKit (Qiagen, Valencia, CA) or from paraffin embedded samples (n = 11) using the InnuPREP DNA Microkit according to the manufacturer's protocol (AnalytikJena, Jena, Germany). As reference DNA samples isolated from peripheral blood mononuclear cells or non-malignant normal breast tissue was used.

FAM or HEX end-labeled primer pairs were used to amplify the di- or tetranucleotide-repeat fragments of 116–280 bp in length (Table S2). The target sequences were amplified by PCR and the PCR products were subsequently separated and detected with a Genetic Analyzer 3130 (Applied Biosystems, Freiburg, Germany). GeneScan software (Applied Biosystems) was used to study the lengths of the allele fragments and fluorescence intensity. The alleles were defined as the two highest peaks within the expected size range. The determination of allelic imbalance (AI) was performed for heterozygous markers by calculating the ratio of the peak heights of the tumor and normal alleles. Ratios of 1.8 or higher were scored as AI.

### Gene expression Analysis of 11p gene in primary tumors and brain metastases

Two different data sets on primary BC were downloaded from GEO (http://www.ncbi.nlm.nih.gov/geo/). The first data set GSE21974 comprising of 32 untreated primary breast tumors without relapse was compared to nine BCBM samples analyzed at our institute. These two datasets, which both were analyzed on the Agilent Whole Human Genome Microarray 4×44 K, were combined, quantile normalized and controlled for systematic differences between the two array groups. Subsequently, differentially expressed genes at the 11p15 locus were selected using the significance analysis of microarrays (SAM) algorithm with a false discovery rate (fdr) of 5% [10]. In addition, a second publicly available data set GSE14020 was also analyzed in order to see if there is a difference in the *PRKCDBP* expression among different primary BC patients with different relapse patterns. The data set consist of primary breast tumors with 22 cases of brain relapse, 20 cases with lung relapse, and 18 cases with bone relapse. The Affymetrix .CEL files were processed using GCRMA. Differentially expressed genes (brain vs. bone relapse and brain vs. lung relapse) were identified by repeated permutation testing using the SAM algorithm using a 5% fdr.

### Quantitative real-time RT-PCR (qRT-PCR) analyses

100 ng total RNA was isolated from fresh frozen tumor tissue using the RNeasy Micro Kit (Qiagen, Hilden, Germany) according to the supplier's instructions using DNase I treatment. The tumor RNA was subsequently reverse transcribed using First Strand cDNA Synthesis Kit (Fermentas St. Leon-Rot, Germany) together with 500 ng of universal human reference (UHR, Stratagene, Agilent technologies, Texas USA). Quantitative real-time RT-PCR (qRT-PCR) analyses were performed on Eppendorf Master Cycler using SYBR Green (Fermentas, St. Leon-Rot, Germany) as fluorescence detection method with the following primers; RPLPO-F: TGAGGTCCTCCTTGGTGAACA, RPLPO-R: CCCAGCTCTGGAGAAACTGC, PRKCDB-F:AGCTCCACGTTCTGCTCTTCA, PRKCDBP-R: GGCGTGAGTGCTACATT CTGA. The analyses were done in triplicates and the mean values were used for each gene. The mRNA levels were normalized to the mRNA level of the ribosomal RPLP0 gene using ΔΔCT-method for quantification [21]. The results, expressed as N-fold differences in target gene expression compared to UHR expression.

### Methylation-specific PCR (MSP)

500 ng of genomic tumor DNA from 13 primary BC patients, 16 BCBM samples were bisulfite treated using the EZ DNA Methylation-Gold Kit (Zymo Research, Freiburg, Germany) and eluted in 16 µl H20. The bisulfite-treated DNA was PCR amplified using primers designed to anneal specifically to the methylated (MF-GAAATAAAAATTTTCGTGATTC, MR-CTTAAAAACGTTTCGCCTTCCG) or un-methylated (UMF-GTTGTGTTAATATAGTTTTTGT, UMR-AAAATCTCTTAAAAACAT TTCA) bisulfite-modified DNA sequence within the gene. Primer sequences for the methylated and the unmethylated allele of *PRKCDBP* were reported previously [22]. 2 µl of modified DNA was amplified in 10 µl reaction mixtures comprising 1 µl of 10× PCR Gold Buffer, 0,5 µl of 10 mM dNTP mix, 0.5 µl of 10 pmol forward and reverse primers, and 0.5 U of AmpliTaq Gold DNA Polymerase DNA (Applied Biosystems, Darmstadt, Germany). MSP was carried out in a thermal cycler at 95°C for 30 s for initial denaturation, followed by 40 cycles (denaturation at 95°C, annealing at gene and methylation specific temperatures, elongation at 60°C for methylated and 54°C for unmetylated PCRs for 20 s) and a final 5 min extension at 72°C. A separation of the PCR products took place in 2% agarose gels, stained with 1 µl of ethidium bromide and visualized under UV spectrophotometry. Bisulfite treated MCF7 and MDA-MB-231 breast cancer cell line DNA were utilized as a positive and negative controls in the analyses. According to the methylation pattern, results were categorized into wild type (WT), heterozygote (HET) and homozygote methylated (MET).

### Statistical Analysis

The relationship between microsatellite markers and clinical factors was examined by means of the χ^2^ -test of independence. Differences between primary BC, BCBM and other types of metastases in relation to allelic imbalance were evaluated with the Wilcoxon rank-sum test. Kaplan-Meier curves were used to estimate survival probability as a function of time, and differences in patient survival were analyzed using the log rank test. Statistical analyses were performed using SPSS Statistical program version 18.0 for Windows (SPSS, Chicago, IL, USA).

## Results

### Loss of 11p in primary and metastatic breast cancer

**Figure 1:**
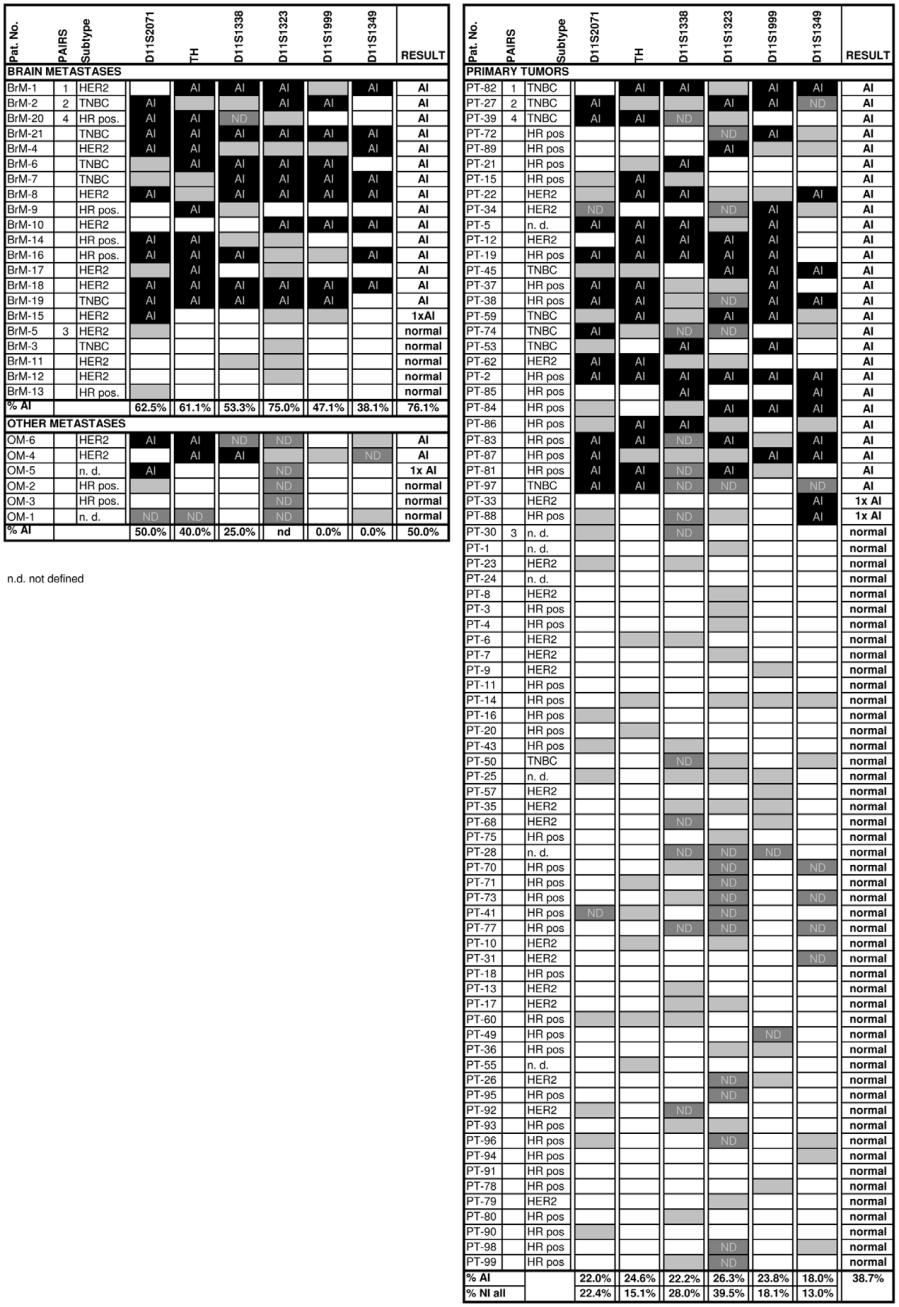
Microsatellite analyses for AI on 11p in primary breast cancers and metastases. Base pair position and the markers used are indicated on the top line. The result for each marker is shown as follows: AI: black; non-informative: light gray; unavailable measurement: dark gray; and informative without changes: white box.

Microsatellite (allelic imbalance, AI) analysis was carried out to verify and to reveal the extent of loss at 11p15.5-p15.3. Six markers spanning an 11.5 MBp region on 11p were analyzed. Altogether 21 BCBM samples, 78 primary BC samples and six samples from other distant metastatic sites were investigated (Figure 1).

The frequency of AI for individual markers varied between 18–26% in the primary tumors and 38–75% in brain metastases (only informative markers taken into account). Marker D11S1323 (11p15.4) had the most AI in both primary BC (26%) and BCBM (75%), whereas the most distal marker D11S1349 (11p15.3) had the fewest AI in all samples (18% and 38%). The degree of non-informative markers ranged between 13–39%, which is in agreement with the literature [18], [19], [23].

Significant differences in the AI frequencies were detected between the primary BC and BCBM. 76.2% of the BCBM were found to be carriers of allelic imbalance (AI) in the 11p region, whereas primary BC tumors showed 37.2% AI at any of the marker in this region (p = 0.002, Table 1). Primary BC with no later history of relapse showed and AI frequency of 36.4% (significant difference compared to BCBM; p = 0.004), and primary tumors from patients with other relapse showed 30% AI (p = 0.010 compared to BCBM). Both primary tumors with later brain metastases as well as three of the six samples from metastatic sites other than brain showed an AI frequency of 50%. Table 1 shows the frequencies and p-values for the different group analyses.

**Table 1:** Frequencies and p-values for AI at chromosome 11p in BCBM, primary tumors and metastases.

		AI all					D11S2017					TH2					D11S1338					D11S1323					D11S1999					D11S1349				
	total	normal		AI		p-value	normal		AI		p-value	normal		AI		p-value	normal		AI		p-value	normal		AI		p-value	normal		AI		p-value	normal		AI		p-value
	n	*n*	%	*n*	%		*n*	%	*n*	%		n	%	n	%		*n*	%	*n*	%		*n*	%	*n*	%		*n*	%	*n*	%		*n*	%	*n*	%	
**BCBM**	21	*5*	23.8	*16*	76.2	-	*6*	37.5	*10*	62.5	-	*7*	38.9	*11*	61.1	-	*7*	46.7	*8*	53.3	-	*3*	25.0	*9*	75.0	-	*8*	50.0	*8*	50.0	-	*12*	60.0	*8*	40.0	-
**Other mets**	6	*3*	50.0	*3*	50.0	n.s.	*2*	50.0	*2*	50.0	n.s.	*3*	60.0	*2*	40.0	n.s.	*4*	80.0	*1*	20.0	n.s.	*nd*		*nd*		n.s.	*5*	100.0	*0*	0.0	n.s.	*3*	100.0	*0*	0.0	n.s.
**primary BC** *	78	*49*	62.8	*29*	37.2	**0.002**	*49*	83.1	*10*	16.9	**0.001**	*49*	76.6	*15*	23.4	**0.004**	*47*	83.9	*9*	16.1	**0.050**	*44*	81.5	*10*	18.5	**0.001**	*49*	77.8	*14*	22.2	n.s.	*54*	84.4	*10*	15.6	n.s.
no relapse	44	*30*	65.2	*16*	34.8	**0.004**	*29*	85.3	*5*	14.7	**0.001**	*33*	80.5	*8*	19.5	**0.003**	*31*	83.8	*6*	16.2	**0.013**	*24*	80.0	*6*	20.0	**0.001**	*28*	77.8	*8*		n.s.	*32*	82.1	*7*	17.9	n.s.
brain relapse	10	*5*	50.0	*5*	50.0	n.s.	*7*	77.8	*2*	22.2	n.s.	*4*	50.0	*4*	50.0	n.s.	*4*	66.7	*2*	33.3	n.s.	*6*	75.0	*2*	25.0	n.s.	*6*	66.7	*3*	33.3	n.s.	*8*	80.0	*2*	20.0	n.s.
other relapse	20	*13*	68.4	*6*	31.6	**0.010**	*13*	81.3	*3*	18.8	**0.029**	*12*	85.7	*2*	14.3	**0.033**	*12*	92.3	*1*	7.7	**0.016**	*14*	87.5	*2*	12.5	**0.001**	*15*	83.3	*3*	16.7	n.s.	*14*	93.3	*1*	6.7	n.s.

* FUP missing for 6 patients.

n.s.: not significant.

When the individual markers were analyzed separately, a statistically significant difference was observed between the BCBM and primary BC without relapse or other relapse than brain for the 4 most telomeric makers (11p15.5-p15.4; all p<0.04). No difference between the two different distant metastasis groups or between BCBM and primary BC with brain relapse could be found.

In four cases matched primary BC and the corresponding BCBM samples were available for the microsatellite analysis. In all cases identical aberration patterns were seen, with three cases showing an AI for the entire region and one case with a normal copy number for 11p15 .

### Clinical significance of AI at 11p in primary BC

Among the primary BC patients occurrence of AI at 11p was significantly associated with high grade (p = 0.050), negative hormone receptor (HR, p = 0.004), and triple-negative (HR and HER2 negative patients; TNBC) status (p = 0.008) (Table 2). None of grade 1 tumors showed an AI, whereas 53% of grade 3 tumors had AI at 11p15. Similarly, only one HR negative patient (11%) did not showed an AI at 11p15, whereas 67% of the HR positive patients were wild type. When the breast cancer patients were classified to the three different subgroups, 89% of TNBC, 47% of HR positive cases, and only 25% of HER2 positive cases showed an AI at 11p15. AI was also more common in higher stages (pT3+ pT4 56% vs. pT1 29%, p = 0.56). A significant association between HR negative status and all individual markers except for the marker D11S1349 was detected (data not shown). In addition, marker D11S2071 was also significantly associated with the TNBC status (p = 0.027). 60% of the TNBC tumors showed an AI at D11S2071, whereas only 21% of the HR positive and 24% of HER2 positive tumors had AI at D11S2071.

**Table 2:** 11p allelic imbalances and association to clinical factors in primary tumors.

	AI all				
	normal		AI		p-value
	*n*	%	*n*	%	
**Histology (n = 78)**					
Ductal	*33*	67.3	*21*	72.4	ns
Lobular	*9*	18.4	*6*	20.7	
others	*7*	14.3	*2*	6.9	
**Age (n = 78)**					
<mean 57.6	*23*	46.9	*16*	55.2	ns
>mean 57.6	*26*	53.1	*13*	44.8	
**Tumor stage (n = 77)**					
pT1	*20*	41.7	*8*	27.6	ns
pT2	*24*	50.0	*16*	55.2	
pT3+4	*4*	8.3	*5*	17.2	
**Lymph node status (n = 77)**					
pNeg	*34*	70.8	*17*	58.6	ns
pNpos	*14*	29.2	*12*	41.4	
**Metastatic status (n = 74)**					
M0	*43*	91.5	*25*	92.6	ns
M1	*4*	8.5	*2*	7.4	
**Grade (n = 77)**					
GI	*3*	6.4	*0*	0.0	**0.05**
GII	*26*	55.3	*10*	34.5	
GIII	*18*	38.3	*19*	65.5	
**Bone marrow status (n = 57)**					
negative	*20*	57.1	*12*	54.5	ns
positiv	*15*	42.9	*10*	45.5	
**Tumor size (n = 76)**					
<mean 2.2 cm	*20*	42.6	*9*	31.0	ns
>mean	*27*	57.4	*20*	69.0	
**Menopausal status (n = 74)**					
perimenop.	*2*	4.3	*0*	0.0	ns
praemenop.	*13*	27.7	*4*	14.8	
postmenop.	*32*	68.1	*23*	85.2	
**Hormone receptor (n = 76)**					
negative	*1*	2.1	*8*	27.6	**0.004**
positive	*46*	97.9	*21*	72.4	
**HER-2 (n = 71)**					
negative	*28*	65.1	*23*	82.1	ns
positive	*15*	34.9	*5*	17.9	
**Subtype (n = 71)**					
HR positive	*27*	62.8	*15*	53.6	**0.008**
TNBC	*1*	2.3	*8*	28.6	
HER2	*15*	34.9	*5*	17.9	
**Ki-67 (n = 71)**					
<20%	*31*	70.5	*15*	55.6	ns
>20%	*13*	29.5	*12*	44.4	
**Relapse (n = 73)**					
no	*28*	59.6	*16*	61.5	ns
yes	*19*	40.4	*10*	38.5	
**Course of the disease (n = 66)**					
alive	*32*	74.4	*20*	87.0	ns
dead	*11*	25.6	*3*	13.0	

HR: hormone receptor.

TNBC: triple-negative tumors (HR negative/HER2 negative tumors).

A total of 66 of the primary BC patients with complete FUP information (R1 and, M1 patients excluded) were eligible for the survival analysis in relation to AI at 11p. AI at 11p was not correlated with relapse or overall survival when all markers were analyzed together. However, AI at the most telomeric marker D11S2071 showed a borderline significant association with earlier relapse compared to patients not showing and AI around this locus (p = 0.052)(Figure S1).

### Differentially expressed genes at 11p15 locus

Array data from primary BC without relapse and BCBM were compared for differentially expressed genes. Using the significance analysis of microarrays (SAM) algorithm altogether 42 genes residing in the 11p15.5-p15.3 region were found to be significantly downregulated among the BCBM samples compared to primary tumors without later relapse (Table 3).

**Table 3:** Differentially expressed genes at 11p between BCBM and primary BC without relapse, bone, or lung relapse.

Agilent ID	Bp position	mean expr. BCBM	mean expr. Primary BC	Fold-change*	GB accesion number	Gene symbol	Brain vrs bone relapse**	Brain vrs lung relapse***
**D11S2071**	235,611							
A_23_P98686	285,251	388	2,949	7.7	NM_025092	ATHL1		
A_24_P287043	299,109	4,014	15,340	3.8	NM_006435	IFITM2	sign	
A_23_P72737	305,209	3,509	40,065	11.4	NM_003641	IFITM1		
A_23_P87545	309,914	6,946	34,681	5.0	NM_021034	IFITM3		
A_23_P84344	395,937	1,633	3,932	2.4	NM_021805	SIGIRR		
A_24_P378019	602,702	1,517	7,071	4.8	NM_004031	IRF7		
A_23_P332960	693,993	1,280	2,675	2.1	NM_001042463	TMEM80		
A_23_P147888	802,818	58,097	147,209	2.6	NM_001004	RPLP2		
A_23_P24784	1,819,013	101	474	4.8	NM_003282	TNNI2		
A_23_P13382	1,869,940	708	2,664	3.7	NM_001013254	LSP1	sign	sign
A_24_P52697	1,973,309	110	385	3.4	NR_002196	H19		
**TH**	**2,148,853**							
A_23_P98671	2,273,543	6	153	26.3	AB029488	C11orf21		
A_32_P123743	2,661,845	1,399	5,023	3.6	NR_002728	KCNQ1OT1		
A_23_P429977	2,826,693	261	702	2.7	NM_000218	KCNQ1		
A_23_P428129	2,861,538	1,276	3,243	2.6	NM_000076	CDKN1C		
A_32_P141488	3,407,086	23	77	3.3	XR_036967	LOC728199		
A_23_P411188	3,643,644	47	172	3.7	NM_020402	CHRNA10		
A_23_P64560	3,803,928	528	1,277	2.4	NM_014489	FRAG1		
A_23_P47691	4,363,144	56	169	3.0	NM_003141	TRIM21		
A_23_P328621	5,492,305	20	90	4.5	NM_145053	UBQLNL		
A_23_P33673	5,590,566	84	373	4.3	NM_001003818	TRIM6		
A_23_P124190	5,611,655	39	170	4.3	NM_130390	TRIM34	sign	
A_23_P203498	5,688,228	73	720	10.0	NM_006074	TRIM22		
**D11S1338**	**5,988,268**							
A_23_P24796	6,189,392	1,514	2,969	2.0	NM_032127	FAM160A2		
A_23_P203475	6,296,845	2,177	6,505	3.0	NM_145040	PRKCDBP	sign	
A_24_P497843	6,372,943	607	1,445	2.4	THC2503819	THC2503819		
**D11S1323**	**6,376,929**							
A_23_P316472	6,549,684	90	243	2.7	NM_144666	DNHD1		
A_23_P98645	6,599,406	54	198	3.7	NM_003737	DCHS1		
A_23_P75850	6,692,420	21	115	5.6	NR_003945	GVIN1		
A_32_P396186	8,590,234	66	261	4.0	NM_014818	TRIM66		
A_23_P203577	8,663,843	38,673	76,549	2.0	NM_000990	RPL27A		
A_23_P24884	8,671,756	341	676	2.0	NM_005418	ST5		
A_23_P105144	9,000,047	40	681	17.2	NM_020974	SCUBE2		
A_23_P321201	9,117,225	682	1,766	2.6	NM_015213	DENND5A		
A_23_P116286	10,485,157	342	761	2.2	NM_001025390	AMPD3		
**D11S1999**	**10,676,524**							
A_23_P98483	10,831,179	1,831	3,769	2.0	NM_021211	ZBED5		
A_32_P118522	10,833,723	17	87	5.3	AI571129	AI571129		
**D11S1349**	**11,709,078**							
A_23_P24843	12,241,805	983	2,879	2.9	NM_014632	MICAL2		
A_24_P931944	12,513,388	570	1,744	3.0	AK128814	AK128814		
A_23_P2032	13,689,673	19	89	4.5	A_23_P2032	A_23_P2032		
A_32_P133072	14,245,756	130	1,198	9.1	NM_006108	SPON1	sign	sign
A_23_P202860	14,856,267	1,424	2,796	2.0	NM_024514	CYP2R1		

* fold change down regulated in brain metastases samples compared to primary breast tumors.

** significantly down regulated genes among primary tumors with brain relapse compared to primary tumors with bone relapse.

*** significantly down regulated genes among primary tumors with brain relapse compared to primary tumors with lung relapse.

In order to further narrow down the possible target gene, primary tumors with different relapse patterns (GSE14020 data set) were also analyzed for differentially expressed genes in the 11p15 region. Patients with later brain relapse were compared to patients with either bone or lung relapse. When primary tumors with later brain relapse were compared to primary tumors with lung relapse only five genes were detected differentially downregulated among the brain relapse patients. Two of these genes (*LSP1* and *SPON1*) were also found significant in the BCBM analysis (Table 3). When the brain relapse patients were compared to the bone relapse patients, altogether 24 genes were detected significantly downregulated in the 11p15 region among the brain relapse patients. Five of these genes (*PRKCDBP*, *IFITM2*, *LSP1*, *TRIM34*, *SPON1*) were identical to those identified significantly downregulated among the BCBM patients (Table 3).

### PRKCDBP expression in BCBM and primary BC samples

**Figure 2:**
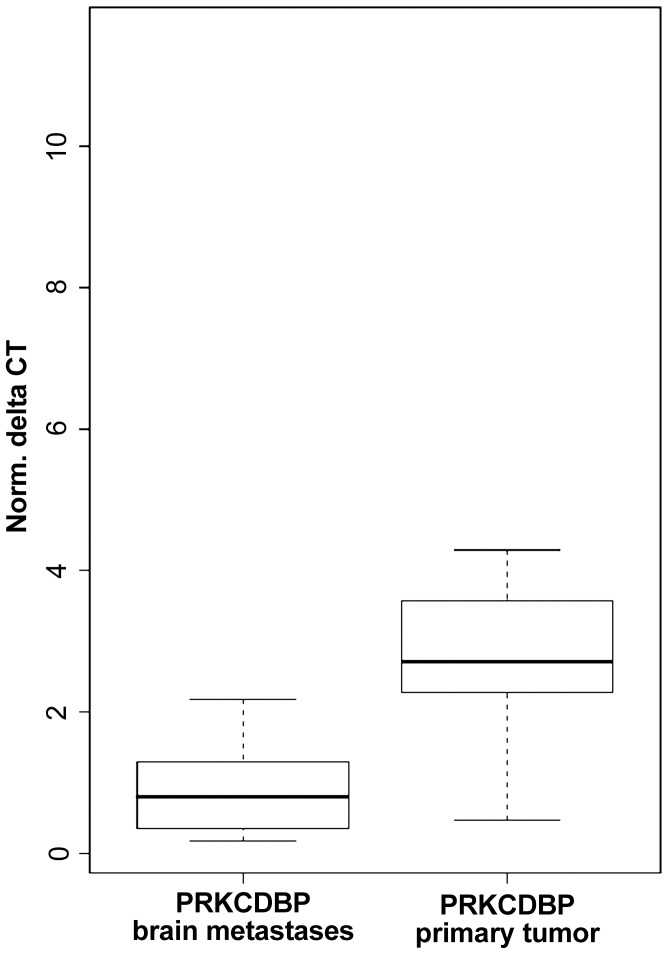
Quantitative real-time RT-PCR results for PRKCDBP expression in BCBM and primary BC patients. Relative PRKCDBP transcript levels were determined by normalization to the reference gene RPLP0 and universal human reference (UHR) using the ΔΔCt method.

The *PRKCDBP* gene located in 11p15 region was chosen for verification analyses by real-time quantitative RT-PCR based on the *in silico* microarray results and because this gene has been indicated to function as a tumor suppressor gene in other epithelial cancers. The *PRKCDBP* mRNA expression was compared between 15 BCBM samples and 23 primary BC samples. The microarray finding could be confirmed, and a statistically highly significant down regulation of *PRKCDBP* was found among the BCBM samples compared to the primary BC (p = 0.001) (Figure 2).

### Methylation analysis of PRKCDBP

Methylation analysis was performed by bisulfite conversion of genomic DNA and methylation-specific PCR (MSP). A methylation of a CpG island in the promoter region of the *PRKCDBP* gene has been previously described which causes a downregulation of the PRKCDBP protein expression [24].

The methylation status of *PRKCDBP* was determined in 16 BCBM and 13 primary BC samples. A homozygous methylation of *PRKCDBP* was detected in six of 16 (38%) of the BCBM samples and in only one (8%) of the primary BC samples. Furthermore, three cases (19%) of BCBM and eight (62%) of the primary BC cases had a heterozygous methylation of *PRKCDBP*, respectively. The methylation status was not statistically significantly associated in either the primary BC or BCBM samples with any clincopathological factor (data not shown).

For one of the homozygously methylated samples expression data was also available. This case showed a silencing of the gene. Among the seven cases with heterozygous methylation, three persons had low expression and four had intermediate or high expression. In addition, two cases with low expression did not show any methylation of the gene, indicating that there are clearly other additional ways to regulate the gene expression of *PRKCDBP*.

## Discussion

In a previous study we compared the array CGH aberration patterns between primary BC and BCBM patients and identified loss off 11p15 to occur significantly more often in the BCBM patients [10]. In this study, we investigated this region in more detail and correlated the findings with clincopathological factors in both primary and metastatic tumors. We detected significant differences in the AI frequencies between the primary BC and BCBM. 76% of the BCBM were found to be carriers of allelic imbalance (AI) in the 11p15 region, whereas only 39% of primary BC tumors showed AI in this region. Interestingly, primary BC samples with later brain relapse had almost as high AI rates as the BCBM samples, whereby samples from patients without relapse or other relapses had significantly lower AI frequencies. The four most telomeric makers (11p15.5-p15.4) were all independently associated significantly with BCBM status, while the two last markers showed lower AI frequencies and no statistically significant association.

In the primary BC samples the most telomeric marker D11S2071 was associated with worse prognosis (borderline significance) and triple negative (TNBC) tumor type. Several studies have shown that ERBB2/HER2 and the basal or TNBC subtypes of breast cancer are the predominant types of breast cancer that metastasize to the brain [25], [26]. Our findings indicate that AI in the telomeric region of chromosome 11p is a marker for both TNBC primary tumors and brain metastasis formation. In primary tumors, 89% of TNBC, 47% of HR-positive cases, and only 25% of HER2-positive cases showed an AI at 11p15. Interestingly, among the BCBM samples loss of 11p15 was not significantly associated with TNBC subtype but was common among all subtypes, indicating that the 11p region could be important for the brain metastasis formation independent of the subtype.

Gene expression profiling identified four genes (*PRKCDBP*, *LSP1*, *TRIM34* and *IFTM2*) within the 11p15 core region (11p15.5-p15.4), which were significantly associated with brain relapse in both primary tumors and in BCBM samples. Whereas IFITM2 and TRIM34 have both been described to be induced by interferon but their role in carcinogenesis has not been studied, both *LSP1* and *PRKCDBP* has been before associated with breast cancer [24], [27]. Interestingly, even though LSP1 is mainly expressed by lymphocytes, neutrophils, and macrophages several genome wide association studies (GWAS) have linked at least two polymorphisms (rs3817198 and rs909116) within the *LSP1* gene with breast cancer risk [27], [28].

We decided to verify the expression of the putative tumor suppressor gene *PRKCDBP* (hSRBC/Cavin-3) as the gene has been recently associated with different types of epithelial cancers [22], [29], [30]. It has been shown that PRKCDBP can induce cell cycle arrest and apoptosis and has an ability to suppress tumor cell growth by blocking p53 function [31]. PRKCDBP has also been shown to function as caveolin adapter molecule that regulates caveolae function [32]. In colorectal cancer *PRKCDBP* expression was recently shown to induce the G1 cell cycle arrest and increased cellular sensitivity to various apoptotic stresses [29]. In addition, PRKCDBP delayed the formation and growth of xenograft colorectal tumors and improved tumor response to TNFa-induced apoptosis, clearly pinpointing *PRKCDBP* as a tumor suppressor gene [29].

The main mechanism behind loss of PRKCDBP expression in cancer seems to be caused by epigenetic mechanisms rather than loss or mutational alterations of the gene itself [30], [31]. The down-regulation of PRKCDBP expression in breast cancer cell lines was associated with hypermethylation of CpG dinucleotides in its promoter region [24]. Furthermore, treatment of breast cancer MCF7 cells with 5′azacytidine a demethylating agent resulted in expression of PRKCDBP, confirming DNA methylation as the mode of inactivation [24]. Indeed, *PRKCDBP* has been shown to be silenced by methylation in different epithelial tumors including ovarian cancer [30], gastric cancer [31] and lung cancer [22]. In gastric cancer loss or reduction of PRKCDBP expression correlated with stage and grade of tumors [31]. In a small study population consisting of five breast tumors Xu et al. detected methylation in three samples [24].

Interestingly also in glioblastoma and neuroblastoma, two central nervous tumors, *PRKCDBP* has been described one of the most frequently methylated genes [33], [34]. In neuroblastoma PRKCDBP expression was also associated with patient outcome [34]. In this study, we could show that *PRKCDBP* expression is significantly more commonly downregulated among BCBM patients compared to primary BC. We further could show that *PRKCDBP* can be donwregulated by methylation in both primary and metastatic breast cancer, but that there are clearly other additional mechanism causing a downregulation of this gene, especially in the brain metastases.

This study identifies a genomic region on chromosome 11p, which might be involved in the development of brain metastases especially among TNBC breast cancer patients. Among the genes located in this region, *PRKCDBP* was frequently downregulated in primary tumors with a high risk of brain metastases. Thus, *PRKCDBP* and other genes in this chromosomal region on 11p might suppress brain metastasis development in breast cancer and could be explored as diagnostic markers or therapeutic targets.

## Supporting Information

Figure S1 **Association of AI at D11S2071 with prognosis in primary BC.** Association of time to relapse with AI was calculated by LOG rank test and illustrated in Kaplan-Meier curves. Continuous line illustrates cases with AI, dotted line normal status.
(TIF)

Table S1 **Clinicopathological characteristics of the patients.**
(XLS)

Table S2 **Primers used for the AI analyses.**
(XLS)
