# Supplementary figures and images for: Clinical Relevance of Loss of 11p15 in Primary and Metastatic Breast Cancer: Association with Loss of PRKCDBP Expression in Brain Metastases
Source: PLoS One. 2012 Oct 31;7(10):e47537. doi: 10.1371/journal.pone.0047537 (PMC3485301; doi:10.1371/journal.pone.0047537)

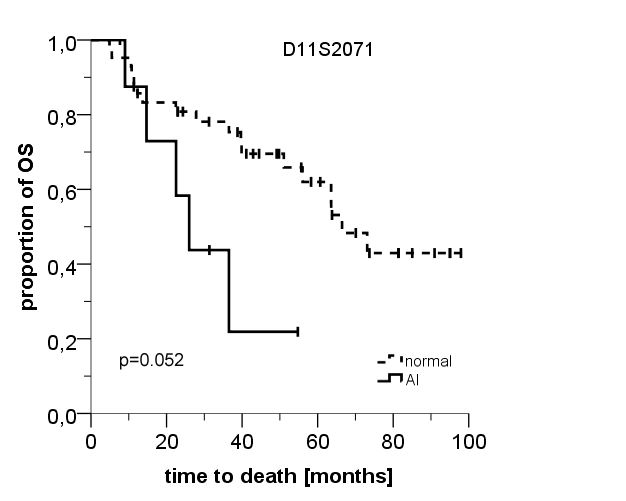

Supplement: Figure S1 — Association of AI at D11S2071 with prognosis in primary BC. Association of time to relapse with AI was calculated by LOG rank test and illustrated in Kaplan-Meier curves. Continuous line illustrates cases with AI, dotted line normal status. (TIF) [file pone.0047537.s001.tif]
